# Epigenetic Alterations and an Increased Frequency of Micronuclei in Women with Fibromyalgia

**DOI:** 10.1155/2013/795784

**Published:** 2013-08-22

**Authors:** Victoria Menzies, Debra E. Lyon, Kellie J. Archer, Qing Zhou, Jenni Brumelle, Kimberly H. Jones, G. Gao, Timothy P. York, Colleen Jackson-Cook

**Affiliations:** ^1^Virginia Commonwealth University School of Nursing, 1100 East Leigh Street, Richmond, VA 23298-0567, USA; ^2^Massey Cancer Center, Virginia Commonwealth University, VA 23298-0037, USA; ^3^Department of Biostatistics, Virginia Commonwealth University, 830 East Main Street, Richmond, VA 23298, USA; ^4^Department of Pathology, Virginia Commonwealth University, P.O. Box 980662, Richmond, VA 23298-0662, USA; ^5^Neodiagnostix, 9700 Great Seneca Highway, Rockville, MD 20850, USA; ^6^Department of Human and Molecular Genetics, Virginia Commonwealth University, P.O. Box 980033, Richmond, VA 23298-0003, USA

## Abstract

Fibromyalgia (FM), characterized by chronic widespread pain, fatigue, and cognitive/mood disturbances, leads to reduced workplace productivity and increased healthcare expenses. To determine if acquired epigenetic/genetic changes are associated with FM, we compared the frequency of spontaneously occurring micronuclei (MN) and genome-wide methylation patterns in women with FM (*n* = 10) to those seen in comparably aged healthy controls (*n* = 42 (MN); *n* = 8 (methylation)). The mean (sd) MN frequency of women with FM (51.4 (21.9)) was significantly higher than that of controls (15.8 (8.5)) (*χ* ^2^ = 45.552; df = 1; *P* = 1.49 × 10^−11^). Significant differences (*n* = 69 sites) in methylation patterns were observed between cases and controls considering a 5% false discovery rate. The majority of differentially methylated (DM) sites (91%) were attributable to increased values in the women with FM. The DM sites included significant biological clusters involved in neuron differentiation/nervous system development, skeletal/organ system development, and chromatin compaction. Genes associated with DM sites whose function has particular relevance to FM included BDNF, NAT15, HDAC4, PRKCA, RTN1, and PRKG1. Results support the need for future research to further examine the potential role of epigenetic and acquired chromosomal alterations as a possible biological mechanism underlying FM.

## 1. Introduction

Fibromyalgia (FM), which affects at least 10 million American adults [1], is a multisymptom condition resulting in not only widespread chronic pain, but also fatigue, sleep disturbances, and morning stiffness. In addition, many patients experience depression, anxiety, and dyscognition [2, 3]. FM has a significant adverse impact on many individuals' physical and mental health [4, 5] and also leads to reduced workplace productivity and increased health care/disability expenses, with the estimated cost of FM on the US economy being reported to be 12–14 billion dollars [1, 6]. While the adverse impact of this condition is indisputable, its etiology remains enigmatic. Due to the lack of clarity for the underlying cause(s) of FM, it poses a diagnostic challenge, often requiring multiple visits by specialists to render a diagnosis [7]. The lack of understanding of the biological basis of this condition also confounds our ability to develop effective interventions and/or monitor disease progression. FM has been suggested to be a complex, multifactorial trait that is influenced by age, gender (frequency is the highest in middle-aged females), and stress/trauma. Despite showing a strong familial aggregation [8–10], attempts to identify genetic factors associated with FM (primarily through polymorphism association studies) have yielded inconsistent results, with some investigators showing associations between FM and specific genes (including, but not limited to, genes for catechol-O-methyltransferase [11–13], serotonin-2A receptor [14, 15], serotonin transporter gene regulatory region [16, 17], dopamine D4 receptor [18], *β*-2 adrenergic receptor [19], gamma-aminobutyric acid receptor subunit beta-3, trace amine-associated receptor 1, interferon-induced guanylate-binding protein 1, regulator of G protein signaling 4, cannabinoid receptor type 1, and glutamate receptor 4 [20]), while others failed to identify a relationship [21–25]. Since a consistent, straightforward association with a gene(s) has not yet been forthcoming, scientists have suggested that the familial influence on FM may more likely reflect a genetic susceptibility to environmental events [21, 26, 27]. For example, Klengel and Binder [28] identified differential methylation for a glucocorticoid response element (the FKBP5 gene) that resulted from the presence of both an “at-risk” allele (polymorphism) and the occurrence of childhood trauma in subjects they studied who had posttraumatic stress disorder. 

Epigenetics, which refers to the process that affects gene expression independent of actual DNA sequence (such as methylation changes, histone alterations, and micro-RNA expression), has enabled scientists to conceptualize the impact of the environment upon one's genes and one's health [29]. Genes can be turned on or off and display variations in their level of expression, in part, due to epigenetic modifications [30]. Thus, epigenetics provides a biological means for understanding the molecular processes of complex biological networks that connect the brain, behavior, and health outcomes [31]. Given the overlap in symptoms and the medical/adverse social histories present in people who have FM, when compared to other conditions that have been shown to be impacted by somatic epigenetic and genetic alterations (such as depression and stress), it is plausible that similar epigenetic mechanisms may underlie the individual variability in the outcome of genetic and emotional inputs for FM.

Knowing that histone and other epigenetic modifications play a key role in establishing and maintaining chromatin structure, it follows that changes in epigenetic profiles, as a consequence of initiating events (such as stress/environmental exposure), could also lead to an increased frequency of somatic chromosomal changes. Indeed, we have shown that stress levels can impact the frequency of acquired chromosomal abnormalities by demonstrating a significantly increased frequency of somatic cell chromosomal instability in adult women who experienced childhood sexual abuse when compared to their identical cotwins who did not experience childhood sexual abuse (quantified using a micronucleus assay) [32]. Further support that chromosomal instability could arise as a downstream effect following perturbations in methylation comes from studies of individuals who have immunodeficiency, centromeric region instability, and facial anomalies (ICF) syndrome, which is an autosomal recessive condition resulting from a mutation in the methyltransferase gene B. People with this condition have an increased frequency of acquired chromosomal abnormalities [33].

An efficient means for quantifying the frequency of acquired (somatic) chromosomal abnormalities is the cytokinesis block micronucleus (CBMN) assay, which provides information regarding the presence of chromosomal errors in somatic cells with minimal influences attributable to *in vitro* selective growth pressures [34]. This technique is less labor intensive than conventional cytogenetic studies but provides results that are in close agreement to those obtained using the “gold standard” of metaphase chromosomal analyses [35]. Micronuclei, which are the primary cytological structures scored in the CBMN assay, are thought to contain chromatin (from one or more chromosomes) that was not incorporated (“lagging” or “lost”) into the daughter binucleates following nuclear division [34]. Micronuclei frequencies have been shown to increase with age and have been associated with a variety of health conditions [36, 37]. However, to date, no investigators have reported the frequency of MN in women with FM. Based on the symptomatology and comorbidities related to this condition, we hypothesized that women with FM will have an increased frequency of acquired epigenetic and chromosomal alterations. To test this hypothesis, we initiated a pilot study to quantify chromosomal instability levels and genome-wide methylation patterns in women having FM and to compare these genetic/epigenetic values to those present in comparably aged, healthy control women.

## 2. Materials and Methods

### 2.1. Study Participant Ascertainment and Specimen Collection

Data for this study were obtained from a subset of participants (*n* = 10), who were randomly recruited by mail from a larger, parent study sample of 64 women diagnosed with fibromyalgia (VCU IRB Protocol number HM12211) (Table 1). In the parent study, the participants completed a two-group randomized, controlled, clinical trial to examine the effect of guided imagery on self-efficacy, perceived stress, pain, fatigue, depression, and neuroendocrine/neuroimmune biomarkers in women with fibromyalgia syndrome [38]. Inclusion criteria for the women having FM were age (at least 18 years old); gender (female); receipt of a physician-confirmed diagnosis of FM based on the 1990 American College of Rheumatology criteria [39]; an ability to understand and sign the consent form; and an ability to understand/complete the study questionnaires. Exclusion criteria for the women in the FM pilot group included the presence of other systemic rheumatologic conditions; being immunocompromised (e.g., diagnosis of HIV/AIDs); receiving corticosteroid treatments; having a major psychiatric or neurological condition that would interfere with study participation, or being pregnant. Each of the study subjects completed a self-report form to provide information regarding age, race/ethnicity, marital status, length of time since diagnosis of fibromyalgia, height and weight for calculating body mass index (BMI), and lifestyle practices such as history of smoking and alcohol use. 

The healthy, comparatively aged control group of women for the MN studies (*n* = 42) were ascertained through their participation in a study of acquired genetic changes associated with normal aging, the latter of which is a twin study [40] (VCU IRB Protocol number 179). The inclusion criteria for this subset of control subjects were gender (female) and age (range from 36 to 69 years old), with all people from the previous study who met the criteria being included as controls for the current study to avoid sampling biases. The control cohort of women included both cotwin pairs (*n* = 30 women or 15 cotwin pairs) and single twins, whose cotwin did not participate in this normal aging study (*n* = 12 women). Due to cost limitations, DNA methylation studies were limited to a subset (*n* = 8) of the control women. This subset of women was randomly selected and included 8 unrelated females (no cotwins). All of the control women self-reported their age, race/ethnicity, and lifestyle practices, such as history of smoking and alcohol use. 

### 2.2. Biological Assays

Following the collection of the blood specimens from the patient and control women, the cells were processed to obtain binucleates for the micronuclei studies and DNA for the methylation studies as described in the following section.

#### 2.2.1. Micronucleus Assay

**Figure 1:**
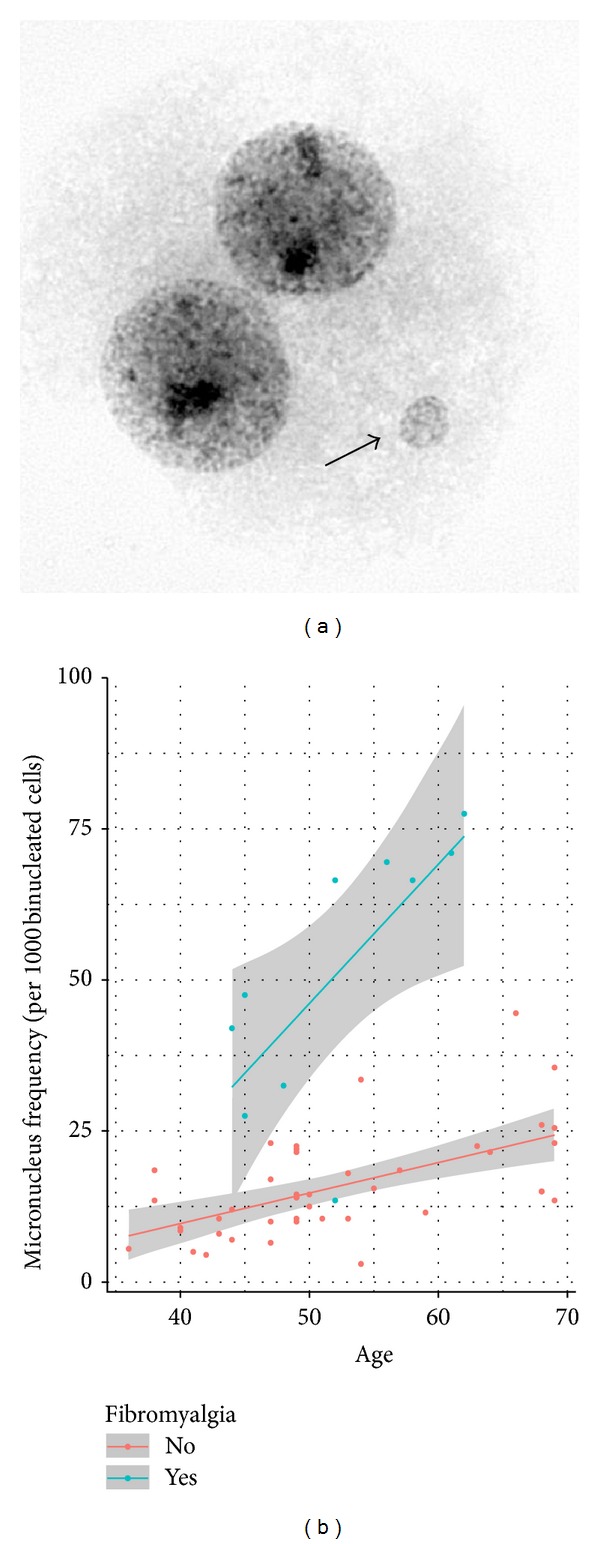
Increased frequency of micronuclei (MN) in women with FM. (a) A representative cell blocked at the cytokinesis phase of mitosis is shown. Karyokinesis has been completed, resulting in 2 daughter binucleates having a single MN (arrow). (b) The frequency of MN (*y*-axis) showed the expected age-related increase (age on *x*-axis) for both controls (Spearman's rho = 0.579) and patients having FM (Spearman's rho = 0.717). The least squares regression line with corresponding 95% confidence interval (grey region) is shown for each group. After controlling for age effects, the frequencies of MN were found to be significantly higher in the women with FM compared to healthy controls (*P* = 1.49 × 10^−11^).

Lymphocytes were collected using Histopaque-1077 (Sigma), stimulated with the mitogen phytohemagglutinin, established in culture, and blocked at cytokinesis according to the protocol of Fenech [35]. Briefly, cytochalasin B (3.0 *μ*g/mL; Sigma, 14930-92-2) was added to the cells 44 hours after culture initiation. At 72 hours, binucleate interphase cells were harvested using standard techniques, which included a 10-minute incubation in hypotonic solution (0.075 M KCl) and serial fixation (three times using a 3 : 1 methanol : acetic acid solution). Slides were made following standard procedures. Micronuclei were visualized following Giemsa staining (4% Harleco Giemsa solution) and identified according to the criteria established by Fenech [35] (Figure 1). The frequencies of micronuclei observed in the cytochalasin-B-blocked binucleated cells of the women with FM and the healthy control women were calculated by averaging the values obtained from two replicate scores (1000 binucleates were evaluated from two independent areas of the slide) for a total of 2000 binucleates that were evaluated per study participant. 

#### 2.2.2. DNA Isolation and Genome-Wide Methylation Assay

Genomic DNA was isolated from whole blood according to standard methods using the Puregene DNA isolation kit (Qiagen). An aliquot (500 ng per study participant) of DNA was then sent to Hudson Alpha Institute for Biotechnology for bisulfite conversion, using standard methods (Zymo Research EZ Methylation Kit) and genome-wide methylation pattern assessment, using the 450 K HumanMethylation Chip, according to the manufacturer's protocol (Illumina). The 450 K HumanMethylation Chip interrogates 485,764 genome-wide targets. Intensity data from the scanned arrays were imported into Illumina's GenomeStudio software and the *minfi* Bioconductor package in the R programming environment to obtain the *β* values for each probe. 

### 2.3. Statistical Analyses

#### 2.3.1. Micronuclei Analyses

To test the hypothesis that women with FM have an increased frequency of acquired chromosomal alterations, the frequency of MN was compared between the women diagnosed with FM and the control group women. Given that a portion of the healthy controls were cotwins, a mixed effect model was used to control for familial relationships. MN frequency comparisons between cases and controls were adjusted for age since several studies have demonstrated increases in MN frequency with age [37, 40–42]. The independent effect of age on MN was evaluated using a Spearman correlation. Additional environmental effects were considered that have previously been shown to influence micronuclei frequencies. These included body mass index, alcohol use, and smoking in the women having FM [40]. However, given that values for body mass index, alcohol use, and smoking were not available for the controls (in a format comparable to those for cases), these variables were only assessed for the women with FM.

#### 2.3.2. Genome-Wide Methylation Analyses

Because the 450 K HumanMethylation assay includes both the Infinium I design (which includes two probes for interrogating a CpG site) and the Infinium II design (which includes only one probe), the GC content was plotted separately by Infinium design type for the fully methylated sample, for which all CpG sites are expected to have consistently high *β* values [43]. Based on these results, probes having a GC content greater than 40 were excluded from further analysis to ensure that the results would not be biased by the “GC” content of the underlying sequence. In addition, since the performance of probes containing single nucleotide polymorphisms (SNPs) can be variable, probes containing SNPs that were present within 10 bases of the target site were also excluded [43]. Because the expression value, *β*, reported for each CpG site represents “proportion methylated” which is constrained to an interval value from 0 to 1, where a *β* of 1 indicates complete methylation and 0 indicates no methylation, the expression values were transformed using the logit transformation [*M* = log⁡(*β*/(1 − *β*)] to promote normality [44]. Prior to the logit transformation, imputation was completed (0.001 for *β* values that were 0 and 0.999 for *β* values that were 1) to avoid nonexistent *M* values. To adjust for the observation that *β* values from the Infinium II designed probes have a compressed range compared to the *β* values from the Infinium I design [43, 45, 46], the peak-correction method was applied to the logit transformed *β* values for the Infinium II designed probes [46].

Statistical analyses were then performed on the peak corrected logit transformed *β* values from the patient and control samples. For each CpG site, differential methylation between specimens collected from women with FM and controls was tested using the moderated *t*-test in the *limma* Bioconductor package [51, 52] in the R programming environment [53]. To adjust for the multiple hypothesis tests, the *P* values were used to estimate the false discovery rate (FDR) following Benjamini and Hochberg's [54] method. The DAVID gene functional classification tool [55] was used to identify biological relationships among the differentially methylated sites. 

## 3. Results

### 3.1. Micronucleus Assay

As expected, the frequency of MN was correlated with age in both the women having FM (*r* = 0.717; *P* = 0.021) and the healthy controls (*r* = 0.579; *P* = 4.79 × 10^−5^) (Figure 1). After controlling for the effect of age and cotwin status, a significantly increased frequency of MN was observed in the women having FM (51.4 (21.9) (mean (sd)) per 1000 binucleates) compared to controls (15.8 (8.5) (mean (sd)) per 1000 binucleates) (*χ* ^2^ = 45.6; df = 1; *P* = 1.49 × 10^−11^) (Figure 1). The increased levels of MN in the women having FM were not significantly correlated with their body mass index (range from 19.44 to 45.70; mean (sd) was 29.52 (7.21); *P* = 0.997), smoking history (4 smokers; 6 nonsmokers; *P* = 0.75), or alcohol use (6 consumers; 4 nonconsumers; *P* = 0.93). To assess if there might be a cumulative biological effect associated with experiencing symptoms associated with FM, we compared MN frequencies for the case subjects (*n* = 10) with the total number of years that had lapsed since these women received their diagnosis of FM. While there was a trend toward a positive correlation between a woman's MN frequency and the number of years since she was diagnosed with FM (ranged from 2 to 19 years), this relationship did not reach significance in this small pilot study (*P* = 0.134) (Table 1).

### 3.2. Genome-Wide Methylation Assay

**Figure 2:**
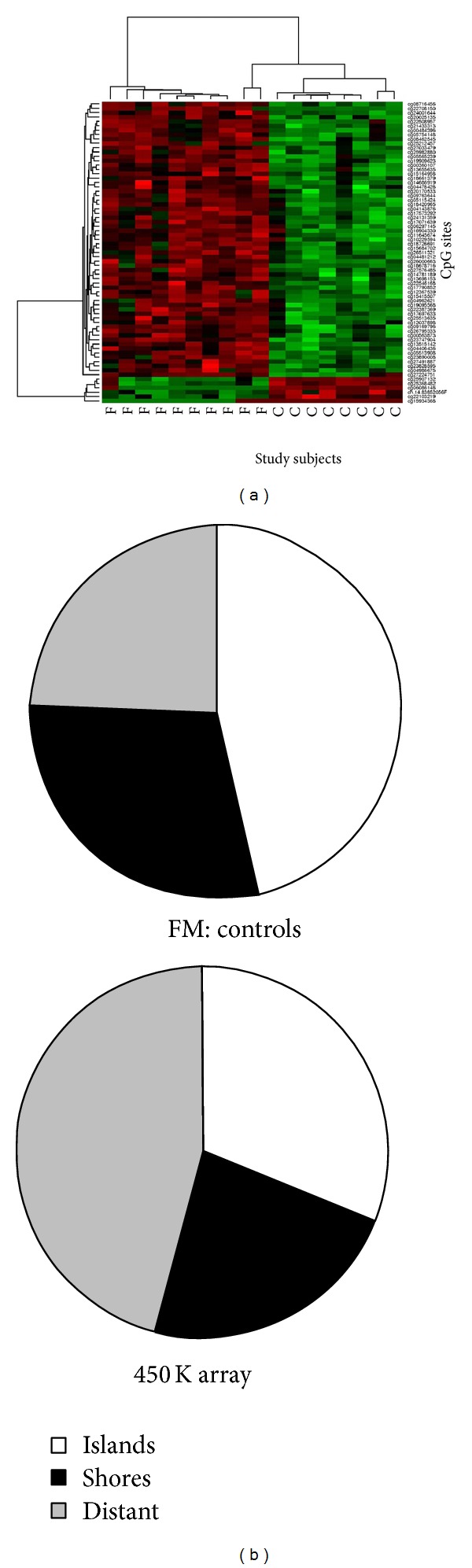
Sites having significantly different methylation patterns in women with FM compared to healthy controls. (a) A heatmap shows the 69 DM sites detected in the study. The women who have FM (F) are grouped on the left of the figure, with controls (C) being grouped on the right. Each row (CpG site) is scaled to represent methylation where lower levels are represented in green while higher levels are represented in red. The majority of significant differences resulted from women with FM (upper left red cluster) having higher levels compared to controls (upper cluster), with a smaller cluster (lower left green) showing lower methylation levels in the cases with FM compared to healthy controls. (b) Pie charts showing the percentage of DM sites according to their relationship to CpG islands. Nearly half (46.4%) of the sites that were significantly different between the women with FM compared to controls were localized to CpG islands (top pie chart), whereas CpG islands account for only about one third (31%) of the sites interrogated on the 450 K HumanMethylation array (bottom pie chart). The proportion of DM sites localized to shores that were recognized in this study (29%) closely paralleled the proportion of sites localized to shores that are interrogated in the array (23%). Sites associated with a location distant to CpG islands accounted for 24.5% of the DM sites identified in this study but accounted for 46% of the total sites interrogated on the array.

After completion of the quality control assessments that were performed to remove any potential biases associated with probe sequence length, probe GC content, and inclusion of SNPs [56], a total of 381,989 CpG sites were retained. From these, a total of 69 sites were determined to be differentially methylated (DM) between the patients who have FM and the healthy controls, with 63 of these DM sites having higher values in the patients with FM and 6 having lower values (Figure 2). These 69 DM sites included CpG islands (46.4%); north shores (20.3%); south shores (8.7%); as well as north (4.3%) and south (1.4%) shelves and sites that were not annotated into the previously noted categories (18.8%). The DM sites were localized to 47 different genes (Table 2), with 3 genes having multiple sites identified (N-acetyltransferase 15 gene (NAT15) had 4 DM sites; DNAJ (Hsp40) homolog, subfamily C, member 17 (DNAJC17) had 2 DM sites; and SLC17A9 and 2 DM sites). An assessment of potential biologically related clusters of DM sites resulted in the recognition of 15 groups, including gene clusters involved in neuron differentiation and nervous system development (Table 3).

## 4. Discussion

While the sample size in this pilot study is small, the MN frequency patterns of both the case and control women showed an age-related increase, which is a finding that is in agreement with the age-related increase that has consistently been reported in larger studies [40, 41]. Interestingly, the mean frequency of MN in the women with FM was *3.26-fold higher* than the level seen in the healthy controls. In comparison, patients who have cancer have been noted to have 1.37- to 3.13-fold higher frequencies of MN when compared to healthy controls [57, 58]. Given that the risk for cancer has been shown to be predicted by MN levels [37, 41, 58], the results of this preliminary data, if confirmed, suggest that MN frequency assessments may be useful for evaluating/diagnosing women with FM. Indeed, recent assessments of MN frequencies in people evaluated from various areas of biobehavioral science have shown increased levels of acquired chromosomal instability (assessed using MN frequencies) in adult women who experienced childhood sexual abuse [32]; patients who have neurodegenerative conditions, such as Alzheimer's disease and Parkinson's disease [59]; and adults with type 2 diabetes and cardiovascular disease [60]. The presence of acquired chromosomal instability, which could lead to somatic tissue mosaicism, has been conjectured to occur as a global biological process that affects many tissues and contributes to the development of several conditions, including (but not limited to) autism, schizophrenia, autoimmune diseases, and Alzheimer's disease [61]. Given that several of these conditions are age related, one could speculate that there may be a “threshold” level of chromosomal instability required for eliciting a biological consequence. Factors contributing to MN formation are multifold and include both genetic [40] and environmental influences [37, 40, 41]. Environmental exposures that have been shown to increase the frequency of MN include, but are not limited to, diet (especially folate deficiency) [41, 62, 63], hormone levels [64], and exposures to substances/occupational hazards [37]. The biological means whereby these genetic/environmental influences lead to acquired chromosomal instability are likely to be varied but have been noted to reflect the chromatin conformation of the chromosomes [65]. One can speculate that alterations in chromatin conformation, which are likely to arise (at least in part) from epigenetic changes, may compromise the ability of the chromosomes to align, attach to mitotic spindle fibers, and/or separate, thereby leading to their increased frequency of abnormalities [66]. In turn, the presence of acquired chromosomal abnormalities could lead to additional epigenetic alterations.

While limited in number, studies performed to assess the effect of methylation on chromosome segregation have consistently shown an increase in the frequency of cytogenic abnormalities associated with perturbations in the methylation status of chromosomes [67]. In this study, it is interesting to note that DM sites were identified for genes having a function related to chromatin compaction and/or segregation (NAT15; HDAC4; UHRF1). For example, DM sites were observed for the NAT15 gene, which is a gene that has been identified to play an important role in normal chromosomal segregation during anaphase [68]. While the results of the genome-wide methylation patterns evaluated in this study are preliminary, it is exciting to note that several of the sites that were DM between the women with FM and controls were localized to genes that have functional relevance to the symptoms seen in patients with FM. Of particular interest was the observation of a significant difference in the methylation pattern of the BDNF gene between patients with FM and controls. The BDNF gene has been noted to play an important neuromodulatory role in pain transduction (eliciting hyperalgesia) [69–71] and has also been recognized as a contributor to learning and memory [72, 73]. A second gene of apparent relevance with which a DM site was associated was the protein kinase C, alpha gene (PRKCA) (Table 3). This gene, which is involved in cell signaling pathways, has been associated with emotional memory formation, posttraumatic stress syndrome, cancer, and aging. A third gene of particular interest that had a DM site is Reticulon 1 (RTN1). RTN1 has been associated with neurological diseases (and cancer) and is thought to influence membrane trafficking in neuroendocrine cells. Overall, genes with which DM sites were associated include (but are not limited to) those having functions in chromatin compaction (NAT15; HDAC4; UHRF1); DNA damage/repair or chromosomal segregation (SOD3; UHRF1; NAT15); muscle contraction (NR4A3; HDAC4; FEZ1; PRKG1; KCNH7); axonal bundling/outgrowth (FEZ1); cell signaling in muscle (NR4A3; PRKG1); neuronal excitability/synaptic transmission (BDNF; BZRAP1; EPHA5; KCNH7); muscle maturation (HDAC4); response to oxidative stress (SOD3); and inflammatory processes (AXL; SH2B2). However, since two of the significant biological clusters that were identified (Table 3) were for polymorphisms and sequence variants, it is important to recognize that this is a pilot study and that a larger number of individuals will need to be evaluated to allow one to determine the extent, if consistently present, of DM on the development or severity of symptoms associated with FM. 

The results of genome-wide methylation studies have provided insight regarding the role of genes and environmental influences for a variety of conditions, with many of these investigations focusing on the areas of cancer and psychiatric conditions [74, 75]. However, the epigenomes of diseases causing chronic pain have been less extensively evaluated. In their study of rheumatoid arthritis, Nakano et al. [76] observed several DM sites between patients who have rheumatoid arthritis and controls. They also identified distinct epigenomic signatures when comparing patterns from patients with rheumatoid arthritis and osteoarthritis. Akin to the results of this pilot study, the findings of their investigation led to the recognition of perturbations in the methylation status of several genes having functions related to the symptoms associated with rheumatoid arthritis. Thus, the use of genome-wide epigenetic assessment seems to be a promising tool for evaluating a broad spectrum of conditions, including those associated with chronic pain.

## 5. Conclusion

In summary, the results of this pilot study suggest that chromosomal instability and alterations in methylation are present in women with FM. If these results can be confirmed, they could provide a basis for improving our understanding of the biological changes leading to the development of FM and may provide a basis for stratifying patients based on their epigenomic and symptom patterns. Moreover, since epigenetic changes demonstrate plasticity [77], the recognition of consistent epigenetic alterations associated with FM could provide a means for developing future therapeutic approaches to reverse these changes, with a goal of alleviating symptoms in people who have FM.

## Figures and Tables

**Table 1:** MN frequencies and other attributes of women with FM.

Participants with FM	Age	Mean number MN per 1000 binucleates	Years since FM diagnosis	Alcohol use	Smoker	Body mass index	Race
1009	48	32.5	2	Yes	No	45.7	Other
1025	52	13.5	3	Yes	Yes	19.4	C^1^
1030	61	71	7	No	No	28.3	C
1038	52	66.5	19	No	No	35.5	C
1042	45	47.5	14	No	No	30.1	AA^2^
1047	62	77.5	19	Yes	No	27.1	C
1052	44	42	1	Yes	Yes	30.5	AA
1058	45	27.5	4	No	No	27.3	AA
1061	58	66.5	2	Yes	Yes	21.1	C
1066	56	69.5	4	Yes	Yes	29.0	AA

FM group mean (sd) (*n* = 10)	48.2 (6.7)	**52.1 (21.9)**				29.52 (7.21)	
Control group mean (sd) (*n* = 42)*	52.0 (9.8)	15.8 (8.5)					

*Individual data not shown.

^1^C: Caucasian (white); ^2^AA: African American (Black).

**Table 2:** Genes associated with sites differentially methylated in patients with fibromyalgia.

Gene name (abbreviation)	Description of function^1^	Location^2^
AXL receptor tyrosine kinase (AXL)	Involved in stimulation of cell proliferation and aggregation; induced by TGF-*β*1/inflammation; and can block cytokine production	19: 41731934
N-Acetyltransferase 15 (NAT15)	Histone acetyltransferase; mediates acetylation of free histone H4; also required for normal chromosomal segregation during anaphase	16: 3507460
Solute carrier family 17, member 9 (SLC17A9)	Vesicular nucleotide transporter involved in exocytosis of ATP in secretory vesicles (synaptic vesicles)	20: 61584072
Brain-derived neurotrophic factor (BDNF)	Involved in the regulation of stress response and in the biology of mood disorders; major regulator of synaptic transmission and plasticity at adult synapses in many regions of the CNS; contributes to several adaptive neuronal responses including long-term potentiation, long-term depression, certain forms of short-term synaptic plasticity, and homeostatic regulation of intrinsic neuronal excitability	11: 27740495
Mahogunin ring finger 1, E3 ubiquitin protein ligase (MGRN1)	Mediates monoubiquitination at multiple sites; plays a role in the regulation of endosome-to-lysosome trafficking.	16: 4714733
Plasma glutamate carboxypeptidase (PGCP)	Involved in hydrolysis of circulating peptides	8: 97657294
Protein kinase C, alpha (PRKCA)	Involved in diverse cell signaling pathways; activated by calcium; associated with cancer; posttraumatic stress syndrome; emotional memory formation; and aging	17: 64787379
Gamma-glutamyltransferase 1 (GGT1)	Function not clear and may vary; has been associated with arterial stiffness and coronary artery disease [47]	22: 24989248
Reticulon 1 (RTN1)	Involved in secretion or membrane trafficking in neuroendocrine cells; associated with neurological diseases and cancer	14: 60097209
NFXL1 nuclear transcription factor, X-box binding-like 1 (NFXL1)	Integral to the nucleus and membrane	4: 47917042
Heat shock 70 kDa protein 8 (HSPA8)	Member of the heat shock protein family, functions as a chaperone and facilitates protein folding	11: 122933028
Polymeric immunoglobulin receptor (PIGR)	Member of the immunoglobulin superfamily that facilitates expression of immunoglobulins A and M; regulated by cytokines	1: 207103660
Benzodiazepine receptor (peripheral) associated protein 1 (BZRAP1)	An adaptor molecule known to regulate synaptic transmission [48]	17: 56401800
Transmembrane protein 91 (TMEM91)	*In vivo* function not clearly established	19: 41882253
Neuron-derived orphan receptor 1 (NR4A3)	Target of *β*-andrenergic signaling in skeletal muscle [49]	9: 102588232
V-set and immunoglobulin domain containing 10 (VSIG10)	*In vivo* function not clearly established	12: 118541722
Potassium voltage-gated channel subfamily H member 7 (KCNH7)	Involved in regulating neurotransmitter release, heart rate, insulin secretion, neuronal excitability, epithelial electrolyte transport, smooth muscle contraction, and cell volume.	2: 163695111
V-set and transmembrane domain containing 2A (VSTM2A)	*In vivo* function not clearly established	7: 54609587
Ephrin type-A receptor 2 (EPHA2)	Protein-tyrosine kinase family member; implicated in mediating developmental events, particularly in the nervous system.	1: 16482553
Patched 2 (PTCH2)	A transmembrane receptor of the patched gene family; may function as a tumor suppressor in the hedgehog signaling pathway; has been associated with several different types of cancer	1: 45297445
Histone deacetylase 4, (HDAC4)	Responsible for the deacetylation of the core histones; gives tag for epigenetic repression; plays important role in transcriptional regulation, cell cycle progression, and developmental events; also involved in muscle maturation through interaction with the myocyte enhancer factors	2: 240044021
ADP-ribosylarginine hydrolase (ADPRH)	Catalyzes removal of mono-ADP-ribose from arginine residues of proteins in the ADP-ribosylation cycle.	3: 119299162
Fasciculation and elongation protein zeta 1 (zygin I) (FEZ1)	Involved in normal axonal bundling and elongation within axon bundles; may also function in axonal outgrowth.	11: 125365478
Superoxide dismutase 3, extracellular (SOD3)	Antioxidant enzyme thought to protect the brain, lungs, and other tissues from oxidative stress.	4: 24801801
Transcription factor AP-2 alpha 2 (TFAP2A)	Transcription factor; activates the transcription of some genes while inhibiting the transcription of others	6: 10420079
Odz, odd Oz/ten-m homolog 3 (ODZ3)	May function as a cellular signal transducer	4: 183370512
Ephrin type A receptor 5 (EPHA5)	Mediates developmental events, particularly in the nervous system; plays a role in synaptic plasticity in adult brain through regulation of synaptogenesis; also mediates communication between pancreatic islet cells to regulate glucose-stimulated insulin secretion	4: 66535145
Suppressor of fused homolog (Drosophila) (SUFU)	Plays a role in pattern formation and cellular proliferation during development; encodes a negative regulator of the hedgehog signaling pathway	10: 104393081
Rh family, C glycoprotein (RHCG)	Functions as an electroneutral and bidirectional ammonium transporter	15: 90039613
DNAJ (Hsp40) homolog, subfamily C, member 17 (DNAJC17)	Heat shock protein homolog	15: 41062113
Autism susceptibility candidate 2 (AUTS2)	Function not fully known; deletions of this gene have been associated with autism and intellectual disability	7: 70158761
Deleted in lymphocytic leukemia, 7 (DLEU7)	*In vivo* function not clearly established	13: 51417846
SH2B adaptor protein 2 (SH2B2)	Involved in multiple signaling pathways; may function as a negative regulator of cytokine signaling; suppresses PDGF-induced mitogenesis: may induce cytoskeletal reorganization via interaction with VAV3	7: 101934892
Alpha-kinase 3 (ALPK3)	Recognizes phosphorylation sites (alpha-helical conformation); may play a role in cardiomyocyte differentiation	15: 85360691
VENT homeobox (VENTX)	May function as a transcriptional repressor and influence mesodermal patterning and hemopoietic stem cell maintenance	10: 135050326
Lymphocyte antigen 6 complex, locus G5C (LY6G5C)	Located in the major histocompatibility complex (MHC) region on chromosome 6; may be involved in signal transduction or hematopoietic cell differentiation	6: 31649619
Primary ciliary dyskinesia protein 1 (PCDP1)	May function in ciliary motility	2: 120301847
Protein kinase, cGMP-dependent, type I (PRKG1)	Involved in signal transduction processes in diverse cell types; plays role in regulating cardiovascular and neuronal functions and in relaxing smooth muscle tone, preventing platelet aggregation, and modulating cell growth	10: 52833610
MAP7 domain containing 2 (MAP7D2)	X-linked imprinted gene that may affect sex-specific brain function and/or sex-dependent neurobiological traits [50]	X: 20134719
Carboxypeptidase M (CPM)	Associated with monocyte to macrophage differentiation; may play role in control of peptide hormone and growth factor activity at the cell surface and in the membrane-localized degradation of extracellular proteins	12: 69346994
Growth differentiation factor 1 (GDF1)/LAG1 longevity assurance homolog 1 (LASS1)	Member of TGF-beta superfamily; may function in regulation of cell growth and differentiation in embryonic and adult tissues and neural development in later embryogenesis; may be involved in aging	19: 18981378
Ubiquitin-like, containing PHD and RING finger domains, 1 (UHRF1)	Recruits a histone deacetylase to regulate gene expression; involved in G1/S transition and functions in the p53-dependent DNA damage checkpoint	19: 4916593
Anoctamin 3 (AN03)	May act as a calcium-activated chloride channel	11: 26353723
Homeobox protein Hox-A7 (HOXA7)	DNA-binding transcription factor that may regulate gene expression, morphogenesis, and differentiation	7: 27196790
Transmembrane protein 44 (TMEM44)	Enriched in the bottom portion of taste buds	3: 194353554
Potassium voltage-gated channel, KQT-like subfamily, member 1 (KCNQ1)	Voltage-gated potassium channel required for repolarization phase of the cardiac action potential; mutations associated with hereditary long QT syndrome 1, the Jervell and Lange-Nielsen syndrome, and familial atrial fibrillation; exhibits tissue-specific imprinting; located in a region associated with the Beckwith-Wiedemann syndrome	11: 2554583
Polymerase I and transcript release factor 1 (PTRF)	Regulates rRNA transcription; thought to modify lipid metabolism and insulin-regulated gene expression	17: 40558063

^1^Functional descriptions obtained from Gene Cards and/or Wikipedia summations and indicated references.

^2^Chromosome: starting nucleotide position.

**Table 3:** Cluster analysis of genes having significantly different methylation patterns in women with FM.

Genetic ontology/keyword term	No. of Genes	*P* value
Anatomical structure development	17	0.0002
System development	16	0.0003
Developmental process	18	0.0008
Multicellular organismal development	17	0.0009
Polymorphism	41	0.0011
Sequence variant	42	0.0012
Domain: fibronectin type III 1, 2	4	0.0045
Glycosylation site	20	0.0047
Neuron differentiation	**6**	**0.0048**
Nervous system development	**9**	**0.0053**
Protein metabolic process	15	0.0067
Autophosphorylation	3	0.0069
Glycoprotein	20	0.0073
Skeletal system development	5	0.0085
Organ development	11	0.0097
